# 2-(1*H*-Benzotriazol-1-yl)-1-phenyl­ethanol

**DOI:** 10.1107/S1600536810011098

**Published:** 2010-03-27

**Authors:** Özden Özel Güven, Meral Bayraktar, Simon J. Coles, Tuncer Hökelek

**Affiliations:** a Department of Chemistry, Zonguldak Karaelmas University, 67100 Zonguldak, Turkey; b Department of Chemistry, Southampton University, Southampton SO17 1BJ, England; c Department of Physics, Hacettepe University, 06800 Beytepe, Ankara, Turkey

## Abstract

In the title compound, C_14_H_13_N_3_O, the benzotriazole ring is oriented at a dihedral angle of 13.43 (4)° with respect to the phenyl ring. In the crystal structure, inter­molecular O—H⋯N hydrogen bonds link the mol­ecules into chains along the *b* axis. Aromatic π–π contacts between benzene rings and between triazole and benzene rings [centroid–centroid distances = 3.8133 (8) and 3.7810 (8) Å, respectively], as well as a weak C—H⋯π inter­action involving the phenyl ring, are also observed.

## Related literature

For general background to the biological activity of benzotriazole derivatives, see: Hirokawa *et al.* (1998 ▶); Yu *et al.* (2003 ▶); Kopańska *et al.* (2004 ▶). For related structures, see: Caira *et al.* (2004 ▶); Katritzky *et al.* (2001 ▶); Özel Güven *et al.* (2008 ▶); Swamy *et al.* (2006 ▶).
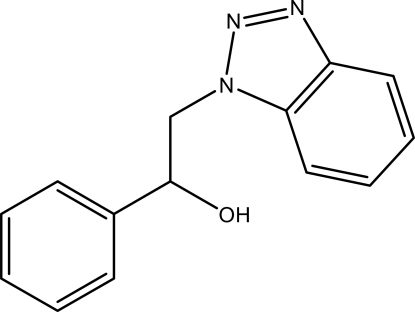


## Experimental

### 

#### Crystal data

C_14_H_13_N_3_O
*M* *_r_* = 239.27
Orthorhombic, 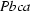

*a* = 11.0731 (3) Å
*b* = 8.6571 (2) Å
*c* = 25.3436 (7) Å
*V* = 2429.5 (1) Å^3^
*Z* = 8
Mo *K*α radiation
μ = 0.09 mm^−1^
*T* = 120 K
0.40 × 0.30 × 0.20 mm

#### Data collection

Nonius Kappa CCD diffractometer
Absorption correction: multi-scan (*SADABS*; Sheldrick, 2007 ▶) *T*_min_ = 0.968, *T*_max_ = 0.981
11997 measured reflections
2772 independent reflections
2447 reflections with *I* > 2σ(*I*)
*R*_int_ = 0.043

#### Refinement

*R*[*F* ^2^ > 2σ(*F* ^2^)] = 0.045
*wR*(*F* ^2^) = 0.105
*S* = 1.06
2772 reflections
215 parameters
All H-atom parameters refined
Δρ_max_ = 0.25 e Å^−3^
Δρ_min_ = −0.23 e Å^−3^

### 

Data collection: *COLLECT* (Nonius, 1998 ▶); cell refinement: *DENZO* (Otwinowski & Minor, 1997 ▶) and *COLLECT*; data reduction: *DENZO* and *COLLECT*; program(s) used to solve structure: *SHELXS97* (Sheldrick, 2008 ▶); program(s) used to refine structure: *SHELXL97* (Sheldrick, 2008 ▶); molecular graphics: *ORTEP-3 for Windows* (Farrugia, 1997 ▶); software used to prepare material for publication: *WinGX* (Farrugia, 1999 ▶) and *PLATON* (Spek, 2009 ▶).

## Supplementary Material

Crystal structure: contains datablocks I, global. DOI: 10.1107/S1600536810011098/im2186sup1.cif

Structure factors: contains datablocks I. DOI: 10.1107/S1600536810011098/im2186Isup2.hkl

Additional supplementary materials:  crystallographic information; 3D view; checkCIF report

## Figures and Tables

**Table 1:** Hydrogen-bond geometry (Å, °)

*D*—H⋯*A*	*D*—H	H⋯*A*	*D*⋯*A*	*D*—H⋯*A*
O1—H1⋯N1	0.85 (2)	1.92 (2)	2.766 (2)	170 (2)
C11—H11⋯*Cg*3^i^	1.01 (2)	2.94 (2)	3.850 (2)	151 (1)

Symmetry code: (i) 
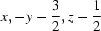
.

**Fig. 1.**
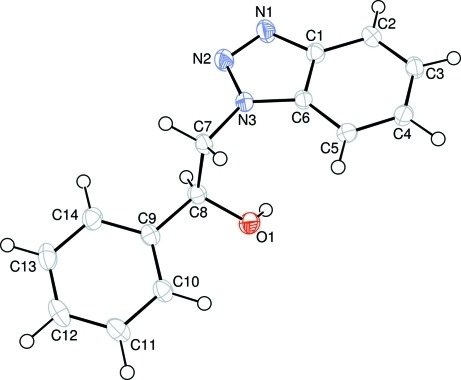
The molecular structure of the title compound with the atom-numbering scheme. Displacement ellipsoids are drawn at the 50% probability level.

**Fig. 2.**
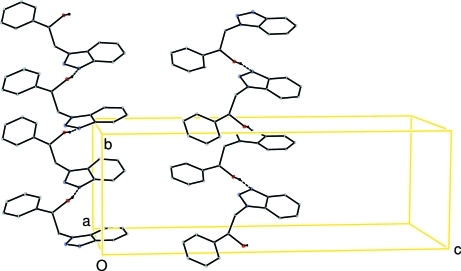
A partial packing diagram. Hydrogen bonds are shown as dashed lines.

## supplementary crystallographic information

### Comment

Azole compounds have important antifungal and antibacterial activities. Benzotriazole derivatives also exhibit a good degree of analgesic, anti-inflammatory, diuretic, antiviral and antihypertensive activities (Kopańska *et al.*, 2004; Yu *et al.*, 2003; Hirokawa *et al.*, 1998). Crystal structures of similar compounds such as 1-phenyl-2-(1*H*-1,2,4-triazol-1-yl)ethanol (Özel Güven *et al.*, 2008), fluconazole (Caira *et al.*, 2004) and benzotriazole ring possessing compounds (Katritzky *et al.*, 2001; Swamy *et al.*, 2006) have been reported. Now, we report herein the crystal structure of the title benzotriazole derivative, (I).

In the molecule of the title compound (Fig. 1), bond lengths and angles are generally within normal ranges. The planar benzotriazole ring system is oriented with respect to the phenyl ring at a dihedral angle of 13.43 (4)°. Exocyclic carbon atoms C7 and C8 are 0.062 (1) and -0.028 (1) Å away from the planes of the benzotriazole and phenyl rings, respectively. So, they are almost coplanar with the adjacent rings.

In the crystal structure, intermolecular O-H···N hydrogen bonds (Table 1) link the molecules into chains along the *b*-axis (Fig. 2), in which they may be effective in the stabilization of the structure. The π–π contacts between the benzene rings and between the triazole and the benzene rings , Cg2—Cg2^i^ and Cg1—Cg2^i^ [symmetry code: (i) 1 - x, 1 - y, -z, where Cg1 and Cg2 are centroids of the rings (C1/C6/N1-N3) and (C1-C6), respectively] with centroid-centroid distances of 3.8133 (8) and 3.7810 (8) Å are also observed in the crystal structure. respectively. A weak C—H···π interaction (Table 1) involving the phenyl ring also occurs.

### Experimental

The title compound was synthesized by the reduction of 2-(-benzotriazol-1-yl)-1-phenylethanone with sodiumborohydride. A mixture of 2-(-benzotriazol-1-yl)-1-phenylethanone (500 mg, 2.10 mmol) and sodiumborohydride (159.5 mg, 4.21 mmol) in ethanol (25 ml) was refluxed for 4 h. After evaporation of the solvent, the mixture was neutralized with dilute HCl, and then refluxed for 30 min. After the mixture was cooled, the solution was alkalinized with dilute NaOH and the resulting precipitate was filtered. The filtrate was extracted with chloroform, then the organic phase was dried and evaporated. The residue was crystallized from ethyl acetate to obtain colorless crystals suitable for X-ray analysis (yield; 216 mg, 43%).

### Refinement

H atoms were located in a difference Fourier map and refined isotropically.

### Crystal data

**Table:**

C_14_H_13_N_3_O	*F*(000) = 1008
*M**_r_* = 239.27	*D*_x_ = 1.308 Mg m^−^^3^
Orthorhombic, *P**b**c**a*	Mo *K*α radiation, λ = 0.71073 Å
Hall symbol: -P 2ac 2ab	Cell parameters from 13033 reflections
*a* = 11.0731 (3) Å	θ = 2.9–27.5°
*b* = 8.6571 (2) Å	µ = 0.09 mm^−^^1^
*c* = 25.3436 (7) Å	*T* = 120 K
*V* = 2429.5 (1) Å^3^	Block, colorless
*Z* = 8	0.40 × 0.30 × 0.20 mm

### Data collection

**Table:**

Nonius Kappa CCD diffractometer	2772 independent reflections
Radiation source: fine-focus sealed tube	2447 reflections with *I* > 2σ(*I*)
graphite	*R*_int_ = 0.043
φ and ω scans	θ_max_ = 27.5°, θ_min_ = 3.1°
Absorption correction: multi-scan (*SADABS*; Sheldrick, 2007)	*h* = −14→14
*T*_min_ = 0.968, *T*_max_ = 0.981	*k* = −10→11
11997 measured reflections	*l* = −32→16

### Refinement

**Table:**

Refinement on *F*^2^	Primary atom site location: structure-invariant direct methods
Least-squares matrix: full	Secondary atom site location: difference Fourier map
*R*[*F*^2^ > 2σ(*F*^2^)] = 0.045	Hydrogen site location: inferred from neighbouring sites
*wR*(*F*^2^) = 0.105	All H-atom parameters refined
*S* = 1.06	*w* = 1/[σ^2^(*F*_o_^2^) + (0.0259*P*)^2^ + 1.6489*P*] where *P* = (*F*_o_^2^ + 2*F*_c_^2^)/3
2772 reflections	(Δ/σ)_max_ < 0.001
215 parameters	Δρ_max_ = 0.25 e Å^−^^3^
0 restraints	Δρ_min_ = −0.23 e Å^−^^3^

### Special details

**Table:**

Geometry. All esds (except the esd in the dihedral angle between two l.s. planes) are estimated using the full covariance matrix. The cell esds are taken into account individually in the estimation of esds in distances, angles and torsion angles; correlations between esds in cell parameters are only used when they are defined by crystal symmetry. An approximate (isotropic) treatment of cell esds is used for estimating esds involving l.s. planes.
Refinement. Refinement of F^2^ against ALL reflections. The weighted R-factor wR and goodness of fit S are based on F^2^, conventional R-factors R are based on F, with F set to zero for negative F^2^. The threshold expression of F^2^ > 2sigma(F^2^) is used only for calculating R-factors(gt) etc. and is not relevant to the choice of reflections for refinement. R-factors based on F^2^ are statistically about twice as large as those based on F, and R- factors based on ALL data will be even larger.

### Fractional atomic coordinates and isotropic or equivalent isotropic displacement parameters (Å^2^)

**Table:**

	*x*	*y*	*z*	*U*_iso_*/*U*_eq_	
O1	0.02061 (10)	0.04949 (12)	0.40376 (4)	0.0279 (2)	
H1	0.090 (2)	0.038 (2)	0.4178 (9)	0.054 (6)*	
N1	0.25101 (12)	0.48299 (16)	0.44171 (5)	0.0300 (3)	
N2	0.18457 (11)	0.44939 (15)	0.40068 (5)	0.0295 (3)	
N3	0.08582 (11)	0.37136 (13)	0.41711 (4)	0.0222 (3)	
C1	0.19546 (12)	0.42532 (16)	0.48602 (5)	0.0224 (3)	
C2	0.22997 (13)	0.43162 (17)	0.53950 (5)	0.0240 (3)	
H2	0.3052 (16)	0.479 (2)	0.5494 (7)	0.029 (4)*	
C3	0.15335 (14)	0.36306 (17)	0.57508 (5)	0.0253 (3)	
H3	0.1736 (15)	0.3631 (19)	0.6119 (7)	0.030 (4)*	
C4	0.04479 (14)	0.29030 (16)	0.55904 (6)	0.0255 (3)	
H4	−0.0089 (16)	0.240 (2)	0.5866 (8)	0.036 (5)*	
C5	0.01041 (13)	0.28261 (16)	0.50680 (6)	0.0228 (3)	
H5	−0.0631 (15)	0.232 (2)	0.4957 (7)	0.028 (4)*	
C6	0.08867 (12)	0.35214 (15)	0.47059 (5)	0.0199 (3)	
C7	0.00031 (13)	0.31427 (16)	0.37785 (5)	0.0229 (3)	
H71	−0.0807 (15)	0.3138 (18)	0.3945 (6)	0.022 (4)*	
H72	0.0019 (15)	0.388 (2)	0.3473 (7)	0.028 (4)*	
C8	0.03379 (12)	0.15050 (16)	0.36022 (5)	0.0216 (3)	
H8	0.1203 (14)	0.1535 (17)	0.3473 (6)	0.021 (4)*	
C9	−0.04730 (12)	0.09913 (16)	0.31533 (5)	0.0214 (3)	
C10	−0.13758 (13)	−0.00987 (17)	0.32359 (5)	0.0248 (3)	
H10	−0.1479 (15)	−0.0567 (19)	0.3588 (7)	0.032 (4)*	
C11	−0.21348 (14)	−0.05425 (19)	0.28253 (6)	0.0303 (3)	
H11	−0.2784 (17)	−0.134 (2)	0.2882 (7)	0.039 (5)*	
C12	−0.19924 (15)	0.00902 (19)	0.23260 (6)	0.0317 (3)	
H12	−0.2518 (18)	−0.023 (2)	0.2038 (8)	0.043 (5)*	
C13	−0.10914 (15)	0.11692 (19)	0.22396 (6)	0.0318 (3)	
H13	−0.0995 (17)	0.162 (2)	0.1892 (8)	0.041 (5)*	
C14	−0.03327 (14)	0.16238 (17)	0.26496 (6)	0.0270 (3)	
H14	0.0301 (16)	0.238 (2)	0.2584 (7)	0.032 (4)*	

### Atomic displacement parameters (Å^2^)

**Table:**

	*U*^11^	*U*^22^	*U*^33^	*U*^12^	*U*^13^	*U*^23^
O1	0.0291 (6)	0.0264 (5)	0.0282 (5)	−0.0010 (4)	−0.0097 (4)	0.0070 (4)
N1	0.0289 (6)	0.0409 (7)	0.0201 (6)	−0.0107 (5)	0.0015 (5)	−0.0021 (5)
N2	0.0311 (6)	0.0367 (7)	0.0206 (6)	−0.0111 (6)	0.0016 (5)	0.0001 (5)
N3	0.0250 (6)	0.0238 (6)	0.0176 (5)	−0.0032 (5)	−0.0009 (4)	0.0004 (4)
C1	0.0222 (6)	0.0248 (7)	0.0202 (6)	−0.0007 (5)	0.0013 (5)	−0.0008 (5)
C2	0.0235 (7)	0.0265 (7)	0.0221 (7)	0.0007 (6)	−0.0024 (5)	−0.0037 (5)
C3	0.0339 (8)	0.0237 (7)	0.0184 (6)	0.0030 (6)	−0.0022 (6)	−0.0002 (5)
C4	0.0332 (8)	0.0219 (7)	0.0213 (7)	−0.0005 (6)	0.0047 (6)	0.0012 (5)
C5	0.0245 (7)	0.0213 (7)	0.0227 (7)	−0.0025 (5)	0.0015 (5)	−0.0003 (5)
C6	0.0227 (6)	0.0191 (6)	0.0178 (6)	0.0017 (5)	−0.0009 (5)	−0.0002 (5)
C7	0.0269 (7)	0.0240 (7)	0.0178 (6)	0.0001 (5)	−0.0051 (5)	−0.0004 (5)
C8	0.0205 (6)	0.0232 (7)	0.0212 (6)	0.0001 (5)	−0.0013 (5)	−0.0002 (5)
C9	0.0212 (6)	0.0233 (7)	0.0197 (6)	0.0048 (5)	0.0004 (5)	−0.0042 (5)
C10	0.0239 (7)	0.0307 (7)	0.0197 (6)	0.0000 (6)	0.0014 (5)	−0.0046 (6)
C11	0.0269 (7)	0.0376 (8)	0.0263 (7)	−0.0032 (6)	−0.0011 (6)	−0.0098 (6)
C12	0.0322 (8)	0.0401 (9)	0.0227 (7)	0.0071 (7)	−0.0065 (6)	−0.0109 (6)
C13	0.0439 (9)	0.0340 (8)	0.0176 (7)	0.0078 (7)	−0.0013 (6)	−0.0024 (6)
C14	0.0317 (8)	0.0274 (7)	0.0218 (7)	0.0015 (6)	0.0033 (6)	−0.0016 (6)

### Geometric parameters (Å, °)

**Table:**

O1—C8	1.4154 (17)	C7—H71	0.992 (17)
O1—H1	0.86 (2)	C7—H72	1.003 (17)
N1—C1	1.3743 (18)	C8—C7	1.5320 (19)
N2—N1	1.3067 (17)	C8—C9	1.5161 (18)
N3—N2	1.3511 (16)	C8—H8	1.012 (16)
N3—C6	1.3659 (16)	C9—C10	1.390 (2)
N3—C7	1.4597 (17)	C9—C14	1.3978 (19)
C1—C2	1.4091 (18)	C10—C11	1.392 (2)
C2—H2	0.962 (18)	C10—H10	0.986 (18)
C3—C2	1.373 (2)	C11—H11	1.005 (19)
C3—H3	0.960 (17)	C12—C13	1.384 (2)
C4—C3	1.417 (2)	C12—C11	1.388 (2)
C4—C5	1.3793 (19)	C12—H12	0.98 (2)
C4—H4	1.014 (19)	C13—H13	0.97 (2)
C5—H5	0.965 (17)	C14—C13	1.393 (2)
C6—C1	1.3974 (19)	C14—H14	0.972 (18)
C6—C5	1.3983 (19)		
			
C8—O1—H1	107.8 (15)	C8—C7—H71	109.9 (9)
N2—N1—C1	108.51 (12)	C8—C7—H72	111.1 (10)
N1—N2—N3	108.76 (11)	H71—C7—H72	110.3 (13)
N2—N3—C6	110.37 (11)	O1—C8—C7	108.63 (11)
N2—N3—C7	118.95 (11)	O1—C8—C9	110.04 (11)
C6—N3—C7	130.55 (12)	O1—C8—H8	111.5 (9)
N1—C1—C2	130.58 (13)	C9—C8—C7	110.30 (11)
N1—C1—C6	108.35 (12)	C9—C8—H8	108.9 (9)
C6—C1—C2	121.07 (13)	C7—C8—H8	107.4 (9)
C1—C2—H2	120.1 (10)	C10—C9—C8	120.79 (12)
C3—C2—C1	116.58 (13)	C10—C9—C14	118.89 (13)
C3—C2—H2	123.2 (10)	C14—C9—C8	120.31 (13)
C2—C3—C4	121.87 (13)	C9—C10—C11	120.60 (14)
C2—C3—H3	119.6 (10)	C9—C10—H10	119.9 (10)
C4—C3—H3	118.5 (10)	C11—C10—H10	119.5 (10)
C3—C4—H4	119.4 (11)	C10—C11—H11	120.9 (11)
C5—C4—C3	122.08 (13)	C12—C11—C10	120.28 (15)
C5—C4—H4	118.5 (11)	C12—C11—H11	118.8 (10)
C4—C5—C6	115.99 (13)	C11—C12—H12	120.1 (12)
C4—C5—H5	122.4 (10)	C13—C12—C11	119.50 (14)
C6—C5—H5	121.6 (10)	C13—C12—H12	120.4 (12)
N3—C6—C1	104.01 (12)	C12—C13—C14	120.49 (14)
N3—C6—C5	133.57 (13)	C12—C13—H13	119.5 (11)
C1—C6—C5	122.41 (12)	C14—C13—H13	120.0 (12)
N3—C7—C8	110.81 (11)	C9—C14—H14	120.0 (11)
N3—C7—H71	107.3 (9)	C13—C14—C9	120.24 (14)
N3—C7—H72	107.4 (10)	C13—C14—H14	119.8 (11)
			
N2—N1—C1—C2	−179.62 (15)	C5—C6—C1—N1	−179.32 (13)
N2—N1—C1—C6	0.05 (17)	C5—C6—C1—C2	0.4 (2)
N3—N2—N1—C1	0.35 (17)	N3—C6—C5—C4	−178.54 (14)
C6—N3—N2—N1	−0.64 (16)	C1—C6—C5—C4	0.0 (2)
C7—N3—N2—N1	−177.00 (12)	O1—C8—C7—N3	65.40 (14)
N2—N3—C6—C1	0.64 (15)	C9—C8—C7—N3	−173.92 (11)
N2—N3—C6—C5	179.36 (15)	O1—C8—C9—C10	13.06 (17)
N2—N3—C7—C8	90.35 (15)	O1—C8—C9—C14	−167.73 (12)
C6—N3—C7—C8	−85.16 (17)	C7—C8—C9—C10	−106.78 (15)
C7—N3—C6—C1	176.45 (13)	C7—C8—C9—C14	72.44 (16)
C7—N3—C6—C5	−4.8 (3)	C8—C9—C10—C11	178.62 (13)
N1—C1—C2—C3	179.42 (15)	C14—C9—C10—C11	−0.6 (2)
C6—C1—C2—C3	−0.2 (2)	C8—C9—C14—C13	−178.95 (13)
C4—C3—C2—C1	−0.3 (2)	C10—C9—C14—C13	0.3 (2)
C5—C4—C3—C2	0.7 (2)	C9—C10—C11—C12	0.5 (2)
C3—C4—C5—C6	−0.5 (2)	C13—C12—C11—C10	−0.2 (2)
N3—C6—C1—N1	−0.42 (15)	C11—C12—C13—C14	−0.2 (2)
N3—C6—C1—C2	179.29 (13)	C9—C14—C13—C12	0.1 (2)

### Hydrogen-bond geometry (Å, °)

**Table:**

*D*—H···*A*	*D*—H	H···*A*	*D*···*A*	*D*—H···*A*
O1—H1···N1^i^	0.85 (2)	1.92 (2)	2.766 (2)	170 (2)
C11—H11···Cg3^ii^	1.01 (2)	2.94 (2)	3.850 (2)	151 (1)

Symmetry codes: (i) ; (ii) *x*, −*y*−3/2, *z*−1/2.
